# Endovascular Management of Arteriovenous Malformations in the Thalamic and Basal Ganglia: A Systematic Review

**DOI:** 10.7759/cureus.76933

**Published:** 2025-01-05

**Authors:** Ali K Alshalchy, Injam Ibrahim Sulaiman, Mohammed Bani Saad, Ali A Bani-Saad, Saleh Saleh, Nooruldeen H. Ali Al-Khafaji, Mustafa Ismail

**Affiliations:** 1 Department of Surgery, University of Baghdad, College of Medicine, Baghdad, IRQ; 2 Department of Surgery, Hawler Medical University, College of Medicine, Erbil, IRQ; 3 Department of Surgery, Al-Kindy Teaching Hospital, Baghdad, IRQ; 4 Department Surgery, University of Baghdad, College of Medicine, Baghdad, IRQ; 5 Department of Surgery, University Of Baghdad, College Of Medicine, Baghdad, IRQ

**Keywords:** arteriovenous malformation, basal ganglia, embolization, endovascular, thalamus

## Abstract

Arteriovenous malformations (AVMs) in the thalamic and basal ganglia present significant challenges due to their deep-seated location and complex vessel architecture. This systematic review outlines the efficacy and outcomes of endovascular management of these lesions. A comprehensive analysis of seven studies including 53 patients revealed high technical success rates, with complete obliteration in 46.7-100% of cases, using advanced embolization agents, such as ethylene vinyl-alcohol copolymer, and precipitating hydrophobic injectable liquid. Adjunctive therapies, mainly stereotactic radiosurgery, further improved results in complex cases. The complications were highly variable, and again, the need for the performance of the technique to be as meticulous as possible was pointed out, tailoring the treatment strategies. This review underlines the potential of endovascular interventions in optimizing outcomes in patients with AVMs in these critical brain regions.

## Introduction and background

Arteriovenous malformations (AVMs) in the thalamus and basal ganglia represent a particularly challenging subset of vascular lesions due to their deep location and proximity to critical neural structures. These malformations account for a small percentage (3-13%) of AVMs in neurosurgical series but are disproportionately associated with poor outcomes, including high rates of hemorrhage, neurological deficits, and mortality [1]. These lesions, therefore, carry a natural history of significantly higher annual hemorrhage rates, as high as 9.8% per patient-year for untreated cases, compared to superficial AVMs [2]. Deep venous drainage and complex angioarchitecture increase this risk further, and conservative management thus remains an option in a few patients. These AVMs, when ruptured, lead to devastating complications in the form of hemiparesis or coma with significant rates of morbidity and mortality [3]. Treatment strategies for AVMs in these eloquent and critical areas have evolved significantly. While stereotactic radiosurgery (SRS) and microsurgical resection are traditional options, they often fall short of addressing the complexities of larger or high-grade AVMs. Recent advances in endovascular techniques, both transvenous and transarterial embolization, using modern agents like copolymer and dimethyl sulfoxide further expand the therapeutic armamentarium and offer the possibility of curative treatment in selected cases [4]. However, even with these newer techniques, pure endovascular management is seldom curative and frequently used as part of multimodal treatment paradigms including in combination with SRS and surgery [1]. This systematic review aims at integrating and critically analyzing the published literature as it relates to endovascular approaches to the management of AVMs involving the thalami and basal ganglia. By assimilating the data regarding outcomes, efficacy, and complication rates, we thus seek to inform the role of endovascular modalities in the management of these formidable lesions and hence identify chinks that should be a focus of active research for the future.

## Review

Methods

Search Strategy

**Figure 1:**
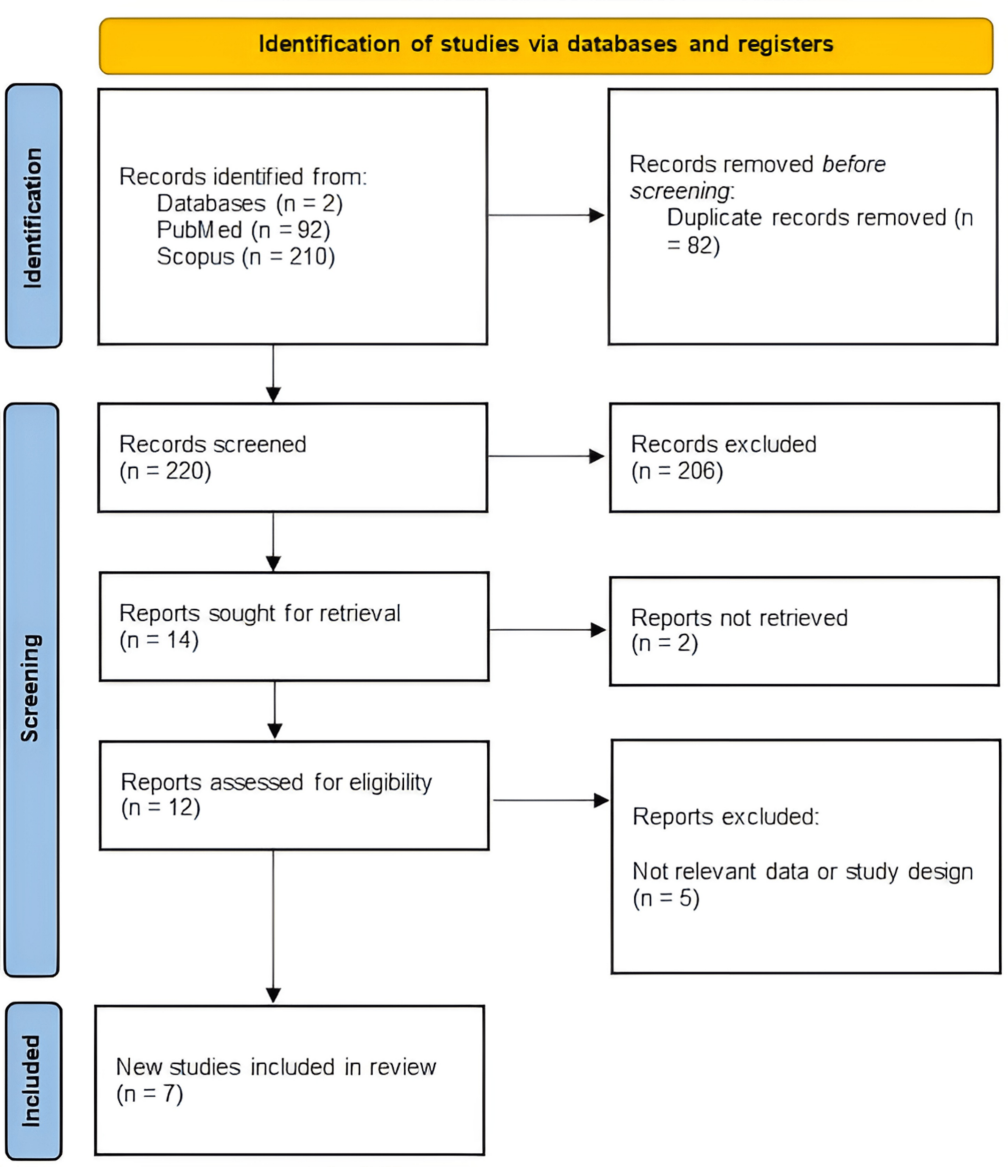
PRISMA flowchart of the included studies PRISMA: Preferred Reporting Items for Systematic Reviews and Meta-Analyses

This systematic review was conducted following the Preferred Reporting Items for Systematic Reviews and Meta-Analyses (PRISMA) guidelines (Figure 1) [5]. A comprehensive literature search was performed across multiple databases, including PubMed and SCOPUS, using a predefined search strategy. The search terms included combinations of keywords and MeSH terms such as "Thalamus", "Thalamic", "Basal Ganglia", "arteriovenous malformation", "AVM", "embolization", and "endovascular". The search strategy was reviewed and refined by an experienced medical librarian to ensure completeness and precision. Additional hand-searching of reference lists from relevant articles was conducted to identify potentially eligible studies not captured in the initial database searches.

Eligibility Criteria

Articles were included if they focused on the endovascular management of thalamic and basal ganglia AVMs, documented clinical outcomes or complication rates, or described technical attributes of embolization in English. All studies involved patients of any age, with a focus on AVMs in either of these deep-seated regions. For a fine view of the current situation regarding clinical practice, this included observational studies, case reports, and their variants. However, specific exclusion criteria were utilized to ensure the quality and relevance of the included studies. These criteria excluded studies that focused on AVMs outside the thalamus and basal ganglia regions. Studies that did not report specific outcomes related to embolization, such as procedural success, obliteration rates, or complications, were also excluded. Additionally, reviews, commentaries, conference abstracts, or editorials that lacked original patient data were not considered. Research involving animal or in vitro studies was excluded, as these did not directly address clinical outcomes. Finally, studies published in languages other than English were not included.

Study Selection

The selection of the study included the screening of titles and their abstracts by two independent reviewers among those retrieved from searches. The screening was made online using the Rayyan platform, which enabled blind assessment and resolution of conflict easily. Articles that were likely to be eligible were full-text retrieved for a second review level. When discrepancies took place between the assessment of one reviewer and the other with regard to the reviewed articles, these were solved by consensus with the inclusion of a third independent reviewer when needed.

Data Extraction

Two reviewers independently extracted data from the included studies using a standardized data collection form. Data collected included study characteristics (author, year, design, and sample size), patient demographics, AVM characteristics (e.g., nidus size, rupture status, and venous drainage), treatment techniques (embolization materials and approaches), and outcomes (technical success, obliteration rates, and neurological outcomes). Any discrepancies in data extraction were resolved through discussion and verification with the original study text.

Risk of Bias Assessment

Methodological quality and the risk of bias for studies included were assessed using specific tools designed for the present study design. The CARE checklists were applied to assess case reports and series for report quality to ensure that these represented articles of high standards from a clinical reporting perspective [6]. The ROBINS-I tool was used for the assessment of bias in nonrandomized studies with respect to participant selection, confounding variables, and outcome measurements [7]. All studies were independently assessed by two reviewers, and their findings were resolved by consensus.

Data Synthesis

The results of the included studies were synthesized qualitatively due to the anticipated heterogeneity in study designs, populations, and outcomes. Where sufficient homogeneity existed, quantitative synthesis was considered using a random-effects meta-analysis model. Stratified analyses were conducted based on AVM characteristics, embolization techniques, and the use of adjunct therapies to explore sources of variability in outcomes.

Results

This systematic review analyzed seven studies focusing on the endovascular management of AVMs located in the thalamus and basal ganglia. The studies included four case reports and three retrospective studies, encompassing a total of 53 patients. These studies were conducted across diverse geographical regions, including South Africa, Japan, China, India, and Peru, reflecting a wide array of patient populations and clinical approaches. The patients ranged in age from 10 to 40 years, with presentations varying significantly in clinical features, AVM characteristics, embolization techniques, and outcomes (Table 1) [8-14].

**Table 1:** Summary of studies on the endovascular management of thalamic and basal ganglia arteriovenous malformations AVM: arteriovenous malformation; ICH: intracranial hemorrhage; DSA: digital subtraction angiography; NCBA: n-butyl 2-cyanoacrylate; PHIL: precipitating hydrophobic injectable liquid

ID	Author, Year	N	Sex (N, %)	Mean Age	Study Design	Location of Study	Clinical Manifestation	Size	Complication	Diagnostic Modality	Embolization Agent	No. of Sessions	Technical Success (%)	Obliteration Rate (%)	Adjunctive Treatment	Follow-up Duration
1	Podlas et al., 1979 [8]	1	Male (100%)	21 years	Case Report	South Africa	Left-sided hemiparesis followed by tremors	Large AVM, right thalamus	None reported	CT, MRI, Angiography	Gelatin foam	1	In this case, technical success was achieved by addressing the residual filling of the AVM after embolization using a surgical approach.	100%	Surgical ligation of feeding vessels	1 month
2	Masayuki et al., 1992 [9]	1	Female (100%)	22 years	Case Report	Japan	Sudden headache, nausea, and consciousness disturbance	AVM, 2 cm, left thalamus	None reported	CT, Angiography	Conjugated Estrogen	1	Progressive size reduction	100% (40 months)	Radiotherapy (30 Gy)	68 months
3	Sun et al., 2010 [10]	15	Male (33.3%), Female (66.7%)	25.1 years	Retrospective	China	ICH (53.3%), hemiparesis (40%)	Mean 3.8 cm (1.5-6 cm)	20%(hemiparesis/hemiplegia due to ischemia)	CT, DSA, Angiography	NBCA, coils, silk sutures	1.6 per patient	≥80% obliteration (33.3%)	46.7% (post-SRS)	Radiosurgery as adjunct	76 months (mean)
4	Zhang et al., 2022 [11]	22	Male Patients: 12 (54.55%), Female Patients: 10 (45.45%)	35.45 years	Retrospective	China	90.9% hemorrhage	The mean AVM size is approximately 3.19 cm	Visual field defect (4.5%)	CT, DSA, MRI	Onyx (82%), Glubran (18%)	1	100% stabilization	86.19%	None required	12 months
5	Lan et al., 2023 [12]	12	Male Patients: 4 (33.33%), Female Patients: 8 (66.67%)	26.33 years	Retrospective	China	100% hemorrhage	The mean AVM size is approximately 3.35 cm	Infarction, brain edema, 1 death (25%)	CT, MRI, DSA	Onyx-18, Glubran-2	A single embolization procedure was performed in 6 patients (50%) and the other 6 cases (50%) were treated in a staged manner with up to three procedures.	58.3% complete obliteration	91.6% (partial + complete)	Radiosurgery for residual lesions	6 months
6	Vargas-Urbina et al., 2023 [13]	1	Male (100%)	10 years	Case Report	Peru	Ruptured thalamic AVM	3.9 mm diameter	None reported	CT, DSA	PHIL 25%	1	100%	100%	None required	6 months
7	Soni et al., 2024 [14]	1	Male (100%)	40 years	Case Report	India	Headache, vision loss	31x21x27 mm	None reported	CT, MRI, DSA	Onyx	1	~75% reduction	Partial	None required	60 days

A total of 302 records were identified through database searches in PubMed (n = 92) and Scopus (n = 210). After removing 82 duplicate records, 220 unique studies were screened. Of these, 206 were excluded during the title and abstract screening for not meeting the inclusion criteria. Full-text retrieval was attempted for 14 reports, but 2 could not be accessed. The remaining 12 reports were assessed for eligibility, and 5 were excluded due to irrelevance in data or study design. Ultimately, seven studies were included in the review.

The quality assessment revealed that, in general, the included studies had good methodological rigor and generally good methodological caliber. The case reports described comprehensive and detailed clinical descriptions by the CARE guidelines in general well-conducted case reports and the retrospective studies fulfilled most of the items in the ROBINS-I checklist with low risks of bias by and large. These studies gave valuable and reliable insights related to the endovascular management of thalamic and basal ganglia AVMs (Tables 2-3).

**Table 2:** Quality assessment of case reports using CARE guidelines CARE: CAse REport

Author, Year	Patient Information	Clinical Findings	Diagnostic Assessment	Therapeutic Interventions	Follow-up Outcomes	Discussion/Conclusions	Overall Quality
Podlas et al., 1979 [8]	Comprehensive	Detailed	Thorough	Well-documented	Reported	Relevant	High
Masayuki et al., 1992 [9]	Comprehensive	Detailed	Thorough	Well-documented	Reported	Relevant	High
Vargas-Urbina et al., 2023 [13]	Comprehensive	Detailed	Thorough	Well-documented	Reported	Relevant	High
Soni et al., 2024 [14]	Comprehensive	Detailed	Thorough	Well-documented	Reported	Relevant	High

**Table 3:** ROBINS-I assessment of the included studies ROBINS-I: Risk Of Bias In Non-randomized Studies - of Interventions

Author, Year	Confounding	Selection of Patients	Classification of Interventions	Deviations from Intended Interventions	Missing Data	Measurement of Outcomes	Selection of Reported Results
Sun et al., 2010 [10]	Low	Moderate	Low	Moderate	Moderate	Moderate	Moderate
Zhang et al., 2022 [11]	Low	Moderate	Low	Moderate	Moderate	Moderate	Low
Lan et al., 2023 [12]	Moderate	Moderate	Low	Low	Moderate	Moderate	Moderate

Clinical Presentation and AVM Characteristics

The clinical presentations of patients with AVMs in the thalamus and basal ganglia were diverse. Hemorrhage was the most common presenting symptom, occurring in a very wide spectrum (53.3% to 100%) of cases in retrospective studies and frequently reported in case reports. Additional symptoms included hemiparesis, sudden headaches, and visual deficits. For example, one study by Sun et al. noted that a wide spectrum of patients (53.3%) of patients presented with intracranial hemorrhage (ICH) while 40% experienced hemiparesis [10]. Similarly, Zhang et al. reported that 90.9% of their patients presented with hemorrhage, with 4.5% experiencing visual field defects [11]. The size of the AVMs also varied significantly. Retrospective studies reported mean AVM sizes ranging from 3.19 cm to 3.8 cm. Smaller lesions were described in case reports such as a 3.9 mm diameter AVM in a pediatric patient reported by Vargas-Urbina et al. [13]. These AVMs were typically located in the thalamus, basal ganglia, or both, underscoring the complexity and criticality of their anatomical positioning.

Embolization Agents and Techniques

Various embolization agents and techniques have been employed in the listed studies, with Onyx being most commonly used, particularly in more recent publications such as Zhang et al. [11] and Lan et al. [12], at 82%-100% of the cases treated. Other agents used included precipitating hydrophobic injectable liquid (PHIL), gelatin foam, N-butyl 2-cyanoacrylate, coils, silk sutures, and conjugated estrogen. Notably, there is a case report of the use of conjugated estrogen by Masayuki et al. [9], which was followed by radiotherapy for the progressive size reduction of the AVM. The number of embolization sessions depended on the size and complexity of the lesion; single-session procedures were done in simpler cases, and staged approaches involving up to three procedures were necessary for more complex AVMs. For example, Lan et al. reported that half of their patients underwent single-session embolization, with the other half requiring staged procedures [12].

Technical Success

The studies reported high technical success rates across different embolization agents and techniques. A 100% technical success rate was noted in all case reports and studies employing advanced embolization agents such as Onyx or PHIL. Sun et al. reported ≥80% obliteration in 33.3% of cases following embolization [10] while Lan et al. reported a combined partial and complete obliteration rate of 91.6% [12].

Obliteration Rates

Obliteration rates were influenced by the use of adjunctive therapies such as SRS. Retrospective studies had overall obliteration rates that ranged from 46.7% to 58.3% when embolization was aided by SRS. Case reports generally demonstrated higher obliteration rates, with Vargas-Urbina et al. achieving 100% obliteration using a transvenous approach with PHIL [13]. Other observations included partial obliteration or reduction in size, as in the 75% reduction reported by Soni et al. after single-session Onyx embolization [14].

Complications

The complication rates varied significantly across studies and were influenced by patient-specific factors, AVM size, and the techniques employed. Retrospective studies, such as those by Sun et al. [10] and Lan et al. [12], reported higher complication rates compared to case reports, likely due to the inclusion of larger sample sizes and more complex cases. Sun et al. documented a 20% complication rate, with hemiparesis and hemiplegia being the most frequent outcomes resulting from ischemic events. Similarly, Lan et al. observed a 25% complication rate, including severe outcomes, such as infarction, brain edema, and one fatality, emphasizing the risks associated with treating large AVMs located in the basal ganglia and thalamus. Case reports highlighted specific complications or their absence in smaller, carefully managed cases. For instance, Vargas-Urbina et al. reported no complications in a pediatric case of ruptured thalamic AVM treated with meticulous imaging guidance and embolization using PHIL 25% [13]. Similarly, smaller AVMs treated with targeted techniques, such as in the cases reported by Podlas et al. [8] and Soni et al. [14], had no reported complications, showcasing the potential of advanced technologies in improving patient safety.

Mortality and morbidity were critical outcomes in larger studies. Lan et al. recorded one fatality, and mobility-related complications, such as hemiparesis and visual field deficits, were common in studies like those by Zhang et al. [11] and Sun et al. [10]. These findings highlight the importance of individualized treatment strategies, the integration of advanced imaging, and embolization techniques to minimize risks. Additionally, the size of AVMs was an important determinant of complications. Retrospective studies reported a mean AVM size of 3.19 to 3.8 cm, with larger lesions being more prone to adverse outcomes. In contrast, smaller AVMs, such as the 3.9 mm lesion reported by Vargas-Urbina et al. [13], were successfully managed without complications. These results underscore the need for meticulous planning and execution in managing AVMs in complex anatomical locations.

Adjunctive Treatments

Adjunctive therapies played a huge role in the optimization of results for larger or complex AVMs. Stereotactic radiosurgery, mainly for residual lesions following embolization, was the most common surgery adjunct. Sun et al. reported 46.7% improved rates of obliteration following the radiosurgery treatment [10]. Equally, in Lan et al. [12] and Zhang et al. [11], radiosurgery was employed as a means of improving obliteration rates with stable courses noted during follow-up.

Follow-Up Outcomes

Follow-up durations varied from as short as 1 month to as long as 76 months. Longer follow-up periods were associated with sustained obliteration and stable neurological outcomes. For instance, Sun et al. observed a mean follow-up of 76 months, reporting no significant recurrence in successfully obliterated AVMs. Similarly, Lan et al. [12] and Zhang et al. [11] demonstrated sustained obliteration and stabilization of symptoms during follow-up periods of 6 to 12 months.

Discussion

From the rudimentary beginnings of endovascular neurosurgery, it has evolved into a cutting-edge specialty. Starting with the first attempts at embolization by Dawbarn in 1904, the introduction of catheter-based angiography, balloon catheters, and newer embolic materials launched this field into state-of-the-art treatments. Major advances in this area include the embolization of AVMs by Luessenhop and Spence in 1960 and the development of the detachable platinum coil by Gugliemi in 1991. Currently, this is a field where innovation and precision go hand in hand in the treatment of complex vascular anomalies, thus redefining neurosurgical care [15]. This systematic review represents a comprehensive assessment of seven studies, including four case reports and three retrospective studies, detailing the progress, challenges, and results of endovascular treatment of AVMs of the thalamus and basal ganglia. The heterogeneity in these studies enables a fine-tuned appreciation of the spectrum of treatment outcomes for diverse clinical scenarios. Technical success was uniformly high across the studies reviewed, reflecting the success of contemporary embolization techniques. For example, Podlas et al. (1979) reported complete technical success in a single case using gelatin foam embolization, achieving stabilization through adjunctive surgical ligation of feeding vessels [8]. Similarly, Masayuki et al. (1992) documented a successful embolization using conjugated estrogen, followed by radiotherapy, which achieved 100% obliteration over a 40-month period [9]. These early case reports demonstrated innovative approaches in the absence of modern embolization agents like Onyx. More recent studies, such as Zhang et al. (2022) [11] and Lan et al. (2023) [12], have shown significant advancements in embolization agents and techniques. Zhang et al. utilized Onyx and Glubran with a 100% technical success rate and an obliteration rate of 86.19%. Lan et al. reported a 58.3% complete obliteration rate with a combination of Onyx-18 and Glubran-2 while achieving a 91.6% combined rate of partial and complete obliteration when staged procedures were employed [12]. These findings present the possibility for staged embolization to further increase the outcomes in larger AVMs or more complex cases. For instance, Zhang et al. (2022) [11] reported an obliteration rate of 86.19% with the predominant use of Onyx while Lan et al. (2023) [12] achieved a 91.6% combined partial and complete obliteration rate using a combination of Onyx and Glubran. These results underline the effectiveness of these agents in both single-session and staged procedures, particularly for larger or more complex lesions. However, the use of these agents is not without risks. Lan et al. (2023) documented a 25% complication rate, including brain infarction, edema, and one fatality [12]. The complications may be attributed to the deep location and critical vascular architecture of the AVMs, which heighten the risk of ischemic events during embolization. While Onyx's non-adhesive nature mitigates some risks, prolonged injection times and inadvertent reflux can still lead to complications. Glubran, on the other hand, requires meticulous handling due to its adhesive properties, which increase the likelihood of catheter entrapment and vascular injury. Moreover, pediatric cases with singular work, such as by Vargas-Urbina et al. (2023) [13], showed 100% obliteration using PHIL through the transvenous approach, hereby illustrating the potential of a new class of embolic agents and techniques for better lesion obliteration even among challenging pediatric patients. Sun et al. (2010) [10] in turn presented a series of 15 patients where they attained ≥80% obliteration in 33.3% of cases; here, too, there was the use of adjunctive SRS, which still bettered the obliteration rates to 46.7%. Similarly, in the case reported by Soni et al. (2024), embolization with Onyx achieved a ~75% reduction in AVM size, underscoring its utility as a debulking tool, particularly in cases where total obliteration is not immediately feasible. Complication rates varied across the studies, with retrospective cohorts reporting higher rates than case reports. Lan et al. (2023) [12] noted a 25% complication rate, which included infarction, brain edema, and one death. Similarly, Sun et al. (2010) [10] reported hemiparesis or hemiplegia due to ischemia in 20% of patients, highlighting the risks inherent in treating AVMs in deep and eloquent regions of the brain. In contrast, complications were notably absent in the single cases reported by Podlas et al. (1979) [8], Masayuki et al. (1992) [9], and Vargas-Urbina et al. (2023) [13], as well as the recent case by Soni et al. (2024). These differences may indicate the individualized attention possible in single-case scenarios or the use of advanced imaging and procedural techniques in more recent studies. In the case reported by Masayuki et al. (1992) [9], radiotherapy (30 Gy) was instrumental in achieving progressive size reduction and eventual obliteration. Of interest, several cases did not require any adjunctive therapies. Zhang et al. (2022) [11] and Vargas-Urbina et al. (2023) [13] reported stabilization and complete obliteration, respectively, without supplementary treatments. This underlines the fact that, in selected cases, embolization alone may already be a curative therapy, especially if modern agents and precise techniques are used. Follow-up durations ranged widely, from 60 days to over 76 months, providing insights into both short- and long-term outcomes. Longer follow-up periods, as in Sun et al. (2010) [10] and Masayuki et al. (1992) [9], demonstrated sustained obliteration and stabilization of neurological symptoms, reflecting the durability of embolization combined with adjunctive treatments. The smaller follow-up duration in, for example, Soni et al. (2024) [14] and Lan et al. (2023) [12], focused essentially on early evidence of procedural success and complication rates. Robert et al. (2017) [16] went so far as to offer a new grading scheme that focused on assessing the endovascular curability of deep-seated AVMs. In this paper, the authors present a review of 134 patients and identify 5 subtypes of AVMs: anterior, lateral, medial, posterior, and midbrain, and the main factors controlling their curability, including type of nidus, pattern of arterial supply, and nature of venous drainage. This classificatory system is designed to improve operative strategy by offering a greater degree of prediction of endovascular outcomes than currently exists [16]. Tuttle et al. (2019) [17] discussed unique challenges created by AVMs in the thalamus and basal ganglia. These AVMs have a higher risk of hemorrhage due to their deep location, small perforating arterial supply, and deep venous drainage. Neuroimaging, especially dynamic MRA, plays a very important role in the identification of the nidus, arterial supply, and venous drainage, which will lead to an accurate diagnosis and treatment planning. The reviewed studies collectively demonstrate significant advancements in the endovascular management of thalamic and basal ganglia AVMs. Early case reports, such as those by Podlas et al. (1979) [8] and Masayuki et al. (1992) [9], showcased innovative approaches using available resources at the time. In contrast, recent studies have benefited from the development of advanced embolization agents like Onyx and PHIL, leading to improved technical success and obliteration rates, as seen in Zhang et al. (2022) [11], Lan et al. (2023) [12], and Vargas-Urbina et al. (2023) [13]. Complications, while still a concern, can be minimized with careful patient selection, precise procedural planning, and the use of advanced imaging techniques. The addition of adjunctive treatments, such as radiosurgery, continues to expand the possibility of complete obliteration and stability in the long term. Finally, the management of these complex lesions is optimally achieved by a multidisciplinary approach tailored to the individual characteristics of both the AVM and the patient. Ongoing refinements in embolization methodology, along with robust long-term follow-up data, will be instrumental in further optimizing results in this challenging subset of AVMs.

Limitations

This review is limited by the small sample sizes and heterogeneous studies included, which reduces the generalizability of the results. Further, several studies did not offer long-term follow-up data, limiting the assessment of sustained outcomes and recurrence rates. Variability in reporting standards and treatment approaches further complicates direct comparisons across studies. Future studies should try to address such gaps with larger, standardized, and longitudinal studies.

## Conclusions

The AVMs of the thalamus and basal ganglia represent a complex group of neurovascular disorders, fraught with significant challenges due to deep location, intricate vessel architecture, and proximity to critical neural structures. This review highlights the potential for endovascular embolization as a cornerstone in the multimodal management of such lesions, with high technical success rates and promising obliteration outcomes, particularly in conjunction with adjunctive therapies like stereotactic radiosurgery. Complications related to a wide variation, thereby emphasizing the need for very careful procedural planning and an individualized approach that can minimize risks. Future research is needed to develop newer and better embolization materials and techniques that could enhance safety and efficacy even further. In addition, the application of advanced imaging modalities and artificial intelligence for accurate preoperative planning and intraoperative navigation is very promising.

## References

[1] Madhugiri VS, Teo MK, Westbroek EM (2019). Multimodal management of arteriovenous malformations of the basal ganglia and thalamus: factors affecting obliteration and outcome. J Neurosurg.

[2] Fleetwood IG, Marcellus ML, Levy RP, Marks MP, Steinberg GK (2003). Deep arteriovenous malformations of the basal ganglia and thalamus: natural history. J Neurosurg.

[3] Murga A, Flores J, Saal G, Osmar Ordinola C, Rodolfo Rodríguez V (2021). Experience of endovascular management of a unruptured thalamus-mesencephalic arteriovenous malformation associated with venous aneurysm in a pediatric patient. Case report. Peruv J Neurosurg.

[4] Kesavan TA, Anees CA, Aparna VE (2021). Arteriovenous malformations in the basal ganglia and thalamus-a rare case of recurrent stroke. IP Int J Med Paediatr Oncol.

[5] Moher D, Liberati A, Tetzlaff J, Altman DG (2009). Preferred reporting items for systematic reviews and meta-analyses: the PRISMA statement. PLoS Med.

[6] Gagnier JJ, Kienle G, Altman DG, Moher D, Sox H, Riley D (2014). The CARE guidelines: consensus-based clinical case report guideline development. J Clin Epidemiol.

[7] Sterne JA, Hernán MA, Reeves BC (2016). ROBINS-I: a tool for assessing risk of bias in non-randomised studies of interventions. BMJ.

[8] Podlas H, Lipschitz R, Allen M (1979). Selective arterial embolization of the brain in an inoperable arteriovenous malformation in the thalamus-a case report. S Afr Med J.

[9] Ezura M, Takahashi A, Yoshimoto T (1992). Successful treatment of an arteriovenous malformation by chemical embolization with estrogen followed by conventional radiotherapy. Neurosurgery.

[10] Sun Y, Lv X, Li Y, Jiang C, Wu Z, Li AM (2010). Endovascular embolization for deep basal ganglia arteriovenous malformations. Neuroradiol J.

[11] Zhang W, Wei H, Tian Q (2022). Efficacy and safety of embolization for arteriovenous malformations of the basal ganglia and thalamus via the transarterial approach. Ann Transl Med.

[12] Lan J, Ma YH, Feng Y, Zhang TB, Zhao WY, Chen JC (2023). Endovascular embolization for basal ganglia and thalamic arteriovenous malformations. Front Neurol.

[13] Vargas-Urbina J, Saal-Zapata G, Durand-Castro W, Rodriguez-Varela R (2023). Transvenous embolization of a ruptured thalamic arteriovenous malformation supplied by the tuberothalamic artery. Surg Neurol Int.

[14] Soni TV, Patel H, Patel MG (2024). Endovascular embolization in a rare case of left basal ganglia large arteriovenous malformation with hydrocephalus: a case report. Asian J Neurosurg.

[15] Richling B (2006). History of endovascular surgery: personal accounts of the evolution. Neurosurgery.

[16] Robert T, Blanc R, Sylvestre P (2017). A proposed grading system to evaluate the endovascular curability of deep-seated arteriovenous malformations. J Neurol Sci.

[17] Tuttle C, Boto J, Martin S, Barnaure I, Korchi AM, Scheffler M, Vargas MI (2019). Neuroimaging of acute and chronic unilateral and bilateral thalamic lesions. Insights Imaging.

